# Retroperitoneal Paraganglioma Treated With Tumor Resection and Replacement of the Inferior Vena Cava

**DOI:** 10.7759/cureus.47160

**Published:** 2023-10-16

**Authors:** Krešimir Đapić, Jasminka Stepan, Maja Pavlović, Vinko Vidjak, Mirko Poljak, Slavko Gašparov, Danko Mikulić

**Affiliations:** 1 Department of Urology, Clinical Hospital Dubrava, Zagreb, HRV; 2 Department of Oncology and Hematology, Children's Hospital Zagreb, Zagreb, HRV; 3 Department of Oncology and Hematology, Children’s Hospital Zagreb, Zagreb, HRV; 4 Department of Diagnostic and Interventional Radiology, University Hospital Merkur, Zagreb, HRV; 5 Department of Surgery, University Hospital Merkur, Zagreb, HRV; 6 Department of Pathology and Cytology, University Hospital Merkur, Zagreb, HRV

**Keywords:** tumor embolization, children, inferior vena cava replacement, retroperitoneal tumors, paraganglioma

## Abstract

Retroperitoneal paragangliomas are tumors of neuroectodermal origin rarely appearing in the pediatric population. We report a case of a large paraganglioma infiltrating the right kidney and inferior vena cava in a 16-year-old boy who initially presented with a right-sided varicocele. Right retroperitoneal paraganglioma was embolized preoperatively, followed by total tumor excision, right nephrectomy, inferior vena cava resection, and reconstruction using a prosthetic vascular graft. Retroperitoneal tumors requiring surgery can successfully be treated by radical resection and replacement of the inferior vena cava in experienced centers.

## Introduction

Retroperitoneal paragangliomas (PGs) are rare primary tumors of neuroectodermal origin arising from extra-adrenal chromaffin cells [1]. Peak incidence for PG is between the ages of 30 and 50, however, some 20% of the cases occur in pediatric patients [2]. While most PGs are functional and present with symptoms of excess catecholamine release, 10%-15% of PGs are nonfunctional and often remain metabolically and clinically silent until reaching considerable size [3]. Approximately 20% of PG are found in the head and neck region, up to 10% in the mediastinal and pericardial localizations, and the remaining 70%-80% arise from infradiphragmatic ganglia. In the abdomen, PG can be found anywhere where sympathetic or parasympathetic ganglia are found, and they most commonly originate from the Zuckerkandl organ near the aortic bifurcation, or retroperitoneally around the kidney, adrenal, and bladder [4]. Surgery remains the mainstay of treatment for PG. Retroperitoneal localization and close relations with major vessels may require challenging surgical approaches.

We report a case of a giant retroperitoneal PG invading the inferior vena cava (IVC) in a 16-year-old boy initially presenting with a right-sided varicocele as the only symptom. The tumor was treated with preoperative embolization and radical surgical excision followed by reconstruction of IVC using a prosthetic vascular graft.

## Case presentation

**Figure 1:**
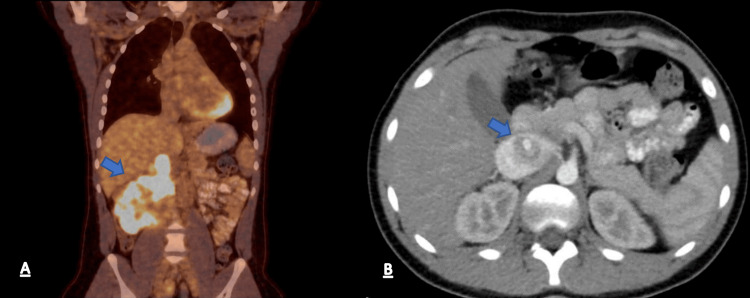
Preoperative PET/CT scan showing (A) increased 18F-FDG uptake involving the right retroperitoneum and inferior vena cava (arrow) and (B) tumor thrombus in inferior vena cava (arrow).

A 16-year-old previously healthy boy was referred to our department after imaging revealed a large retroperitoneal tumor. Renal ultrasound and doppler was initially performed during the work-up of a right-sided varicocele in an otherwise asymptomatic patient. A large, hypervascular heterogenic mass was found in the right retroperitoneum. Magnetic resonance imaging (MRI) showed a vascular tumor of a mixed solid/cystic nature, measuring 14x12x8cm, compressing the lower pole of the right kidney and displacing the kidney laterally. Infiltration of the inferior vena cava (IVC) and an intraluminal tumor thrombus was seen ranging from the level of the right renal vein down to the IVC bifurcation. 18F-FDG PET/CT imaging showed a solid-cystic lesion with an increased 18F-FDG uptake involving the right retroperitoneum and IVC, indicating malignant disease with a probable origin in the IVC (Figures 1A, 1B).

**Figure 2:**
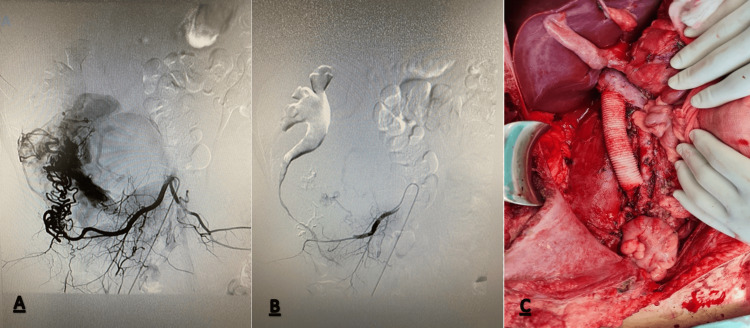
Preoperative embolization photograph showing (A) lumbar artery feeding the tumor and (B) after the embolization. (C) Intraoperative photograph after tumor resection and inferior vena cava reconstruction.

All of the biochemical parameters including catecholamine values and tumor markers were within the normal range. Echocardiography was unremarkable. Due to the hypervascular nature of the tumor, a preoperative percutaneous biopsy was not attempted. The decision was made to operate and, before surgery, preoperative embolization of the tumor was done in order to minimize intraoperative bleeding. Selective angiography of the feeding arteries was performed through the right femoral artery. The tumor was found to be feeding mainly from the lumbar arteries arising from the distal aorta, and from the right renal artery. The main feeding lumbar artery was embolized while the renal artery was not, in order to prevent acute ischemia of the right kidney and possible complications before definitive surgery (Figure 2A, 2B). The patient underwent surgery through a midline laparotomy two days after tumor embolization.

During the operation, tumor was dissected from the pancreaticoduodenal complex, aorta and the right iliac vessels, followed by en-bloc resection including right kidney, right adrenal gland and the IVC from the bifurcation up to the origin of the left renal vein (LRV). The IVC was reconstructed using a 20 mm Polytetrafluoroethylene (PTFE) graft with the upper anastomosis incorporating the infrahepatic cava and the left renal vein (Figure 2C). Paraaortic, inter-aortico-caval and iliacal lymphadenectomy was done as well. Postoperative course was uneventful and the patient was discharged home on postoperative day 10.

**Figure 3:**
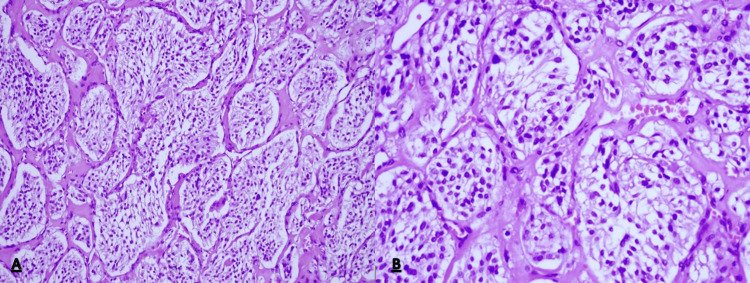
Histopathological features showing the tumor consists of poligonal cells arranged in small nests (Zellballen) separated by delicate fibrovascular stroma (H&E staining, (A) x200, (B) x400).

**Figure 4:**
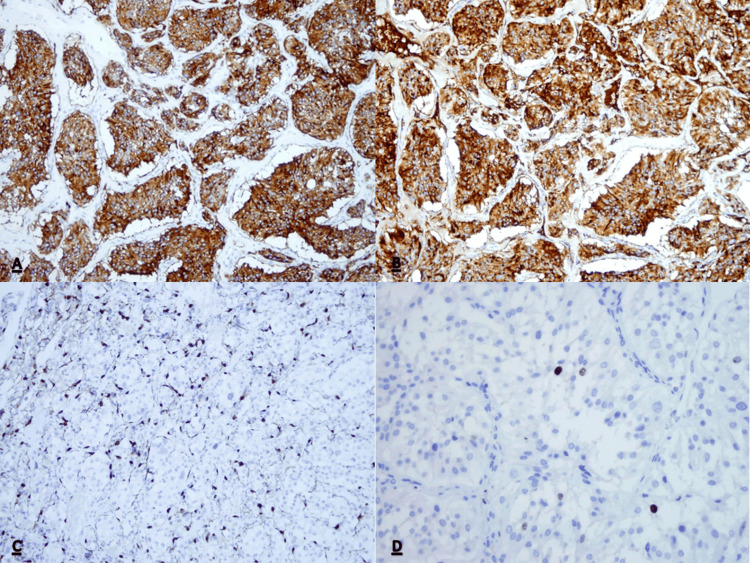
Immunohistochemical features. (A) Strong positive granular cytoplasmic reaction for Synaptophysin (200x). (B) Strong positive granular cytoplasmic reaction for Chromogranin (200x). (C) Positive reaction for S100 in sustentacular cells, situated around the nests of tumor cells (200x). (D) Ki67 proliferative activity in approximately 2% cells (400x).

Histological examination revealed a brownish tumor near the lower pole of the right kidney infiltrating the IVC and the right ureter. On microscopy, tumor cells were arranged in nest-like patterns surrounded by fibrovascular stroma with a characteristic Zellbalen architecture in haematoxilin and eosin staining. The cells had a granular, eosinophyllic cytoplasm and deep staining nuclei. Immunohistochemistry showed positive synaptophysin, chromogranin A and S 100 staining. No mitoses were present, and expression of Ki 67 was 2%. No tumor was found in the regional lymph nodes. Based on the above findings, the histological diagnosis of PG was made (Figures 3A, 3B, 4A-4D).

Six months after surgery, follow-up imaging (MRI and 18F-FDG PET/CT) showed no signs of tumor recurrence or distal metastasis.

## Discussion

Neurogenic tumors are the most common primary retroperitoneal tumors in children. While neuroblastoma is the most common, PGs, originating from extra-adrenal chromaffin cells, uncommonly appear in the retroperitoneum. When located in the adrenal glands, they are called pheochromocytomas. Retroperitoneal PGs usually arise from paraganglia which are part of the sympathetic chain ranging along the aorta. The majority are found near the organ of Zuckerkandl, in the region of the aortic bifurcation and the origin of the inferior mesenteric artery [5]. They are more often found in older children and about 40% are hormonally active, releasing catecholamines [6]. Less than 50% are malignant and due to the similarity in histological appearance to the benign PG, often only the follow-up will reveal the true nature of the tumor. The most common sites of metastasis are regional lymph nodes, bone, liver, and lung. PG can be associated with hereditary disorders like multiple endocrine neoplasia, von Hippel-Lindau disease, neurofibromatosis type 1, and a number of genetic mutations [7]. Retrospective analyses have shown five-year overall survival after surgical resections ranging from 73% to 91%, with high survival rates correlating with the absence of metastasis [8,9].

Histologically, PGs are usually composed of round and oval tumor cells arranged in nests (zellbalen) within the vascular stroma and peripheral sustentacular cells. Nuclear atypia may be present; however, mitoses are rare and there is usually no necrosis. Immunohistochemistry has a substantial role in the confirmation of the diagnosis. Synaptophysin and chromogranin are specific for the presence of tumor cells while sustentacular cells stain positive for anti-S100 antibodies [10]. All of these were positive in our patients. The expression of Ki-67 was low, and analyses had shown that high expression of Ki-67 correlates with metastatic disease [11].

Symptoms in catecholamine-secreting tumors range from headache, palpitation, excessive sweating, and paroxysmal hypertensive crisis to cardiomyopathy and myocardial infarction [12]. Large tumors can present themselves with back pain or a palpable mass. In our case, isolated right-sided varicocele was the only symptom. While usually found on the left, right-sided varicocele always raises suspicion for venous obstruction and an increase in venous pressure. Hence, neoplasm needs to be ruled out either using ultrasound or cross-sectional imaging methods. Wilms’ tumor invasion of the renal vein and the IVC is a typical cause of a secondary varicocele in the pediatric population [13].

Preoperative embolization is routinely performed for cervical PGs. It is reported to reduce intraoperative bleeding, surgical time, and the incidence of postoperative complications [14]. The role of percutaneous embolization in PG is not clear due to paucity of data. One of the concerns is the possible post-intervention release of cathecolamines and the subsequent risk of hypertensive crisis in functioning PG [15].

Retroperitoneal tumor resection with concomitant IVC resection and replacement is routinely performed in adults in experienced centers, mainly for retroperitoneal sarcomas and renal malignancies. When en bloc surgical resection of the tumor and vena cava is required for radical tumor clearance, IVC reconstruction can be performed with low morbidity, low mortality, and excellent clinical results [16]. On the other hand, there have been only a handful of reports in pediatric patients despite the relative frequency of retroperitoneal neoplasms in children [17]. Thanks to the extensive collateral formation through the retroperitoneal and azygos systems, infrarenal IVC can often be safely ligated with adequate venous drainage from the lower body. In our patient, resection of a rich collateral retroperitoneal venous network was necessary in order to achieve complete tumor resection. Also, the IVC was involved with the tumor up to the origin of the left renal vein and ligation of the IVC at this level would impede venous flow through the LRV. It is often argued that the LRV can be safely ligated as long as there are collateral pathways through the left adrenal and testicular veins, however, after the right nephrectomy, we felt it was safer to reimplant the LRV into the neo-cava in order to preserve optimal function of the left kidney. As to the choice of graft for IVC reconstruction, an autologous graft is always a preferable option because of better results in terms of patency, resistance to infection, and future growth [18]. The only published pediatric case of caval resection and reconstruction for resection of a PG that we have found in the literature describes caval reconstruction using autologous veins [19]. However, due to the large size of the defect, prosthetic replacement was the only option for our patient. PTFE prostheses are the type that is usually recommended in the literature since they provide the best results in terms of the length of the missing segment and resistance to intra-abdominal compression [20].

## Conclusions

Radical excision is the treatment option of choice for most retroperitoneal tumors in children. This may require challenging surgical approaches including reconstructions of major retroperitoneal vessels, which can be performed safely in experienced centers.
